# Triglycerides are a predictive factor for arterial stiffness: a community-based 4.8-year prospective study

**DOI:** 10.1186/s12944-016-0266-8

**Published:** 2016-05-18

**Authors:** Xiaona Wang, Ping Ye, Ruihua Cao, Xu Yang, Wenkai Xiao, Yun Zhang, Yongyi Bai, Hongmei Wu

**Affiliations:** Department of Geriatric Cardiology, Chinese PLA General Hospital, Fuxing Road #28, Beijing, 100853 China

**Keywords:** Triglycerides, Carotid–femoral pulse wave velocity, Carotid–radial pulse wave velocity

## Abstract

**Background:**

Epidemiological studies have disclosed an independent effect of triglycerides on coronary heart disease despite achievement of low-density lipoprotein cholesterol goals with statin therapy. Arterial stiffness has been increasingly recognized as a strong predictor of cardiovascular disease and atherosclerotic disease. The association between triglycerides and arterial stiffness is not well characterized. We aimed to determine the relationship between triglycerides and arterial stiffness in a community-based longitudinal sample from Beijing, China.

**Methods:**

We related levels of plasma TGs to measures of arterial stiffness (carotid–femoral pulse wave velocity [PWV] and carotid–radial PWV) in 1447 subjects (mean age, 61.3 years) from a community-based population in Beijing, China.

**Results:**

After a median follow-up interval of 4.8 years, multiple linear regression analysis revealed that TGs were independently associated with carotid–femoral PWV (β = 0.747, *P* < 0.001) and carotid-radial PWV (β = 0.367, *P* = 0.001). In the group older than 65 years, the association between baseline TG levels and follow-up carotid–femoral PWV (β = 1.094, *P* = 0.001) and carotid-radial PWV (β = 0.524, *P* = 0.002) were strengthened. In forward stepwise multivariate logistic regression analysis, every SD increase in TGδ was associated with a 1.296-increased likelihood of the presence of carotid–femoral PWVδII (OR [per SD increase in TGδ]: 1.296; 95 % CI: 1.064 ~ 1.580; *P* = 0.010) in Model 2, whereas the relationship between TGδ and carotid-radial PWVδII disappeared. In addition, the relationship was strengthened between TGδ and the presence of carotid–femoral PWVδII (OR 1.526, 95 % CI: 1.088–2.141, *P* = 0.014) in the group older than 65 years but not carotid-radial PWVδII. No association was noted in subjects younger than 65 years.

**Conclusions:**

Lower triglyceride levels were significantly associated with decreases in carotid–femoral PWV, indicating that achieving low TG levels may be an additional therapeutic consideration in subjects with atherosclerotic disease.

## Background

Patients with cardiometabolic abnormalities remain at high risk of cardiovascular events when low-density lipoprotein cholesterol (LDL-C) goals are obtained. This residual risk is partially due to high TG levels despite achievement of LDL-C goals with statin therapy. Epidemiological studies have disclosed an independent effect of triglycerides (TGs) on coronary heart disease (CHD) events in the presence of lower levels of high-density lipoprotein cholesterol (HDL-C) [1, 2], higher levels of LDL-C [1, 2] and T2DM [3, 4]. A decrease in initially elevated TG levels was associated with a decrease in CHD risk compared with stable high TG levels [5].

Arterial stiffness has been recognized as a strong predictor of subclinical vascular disease as well as cardiovascular mortality [6–9]. Great emphasis has been placed on the role of arterial stiffness, which can be noninvasively assessed via the measurement of pulse wave velocity (PWV) [10, 11]. Carotid-femoral PWV is the proven “gold standard” for arterial stiffness given the largest amount of epidemiological evidence for its predictive value for CHD [12].

Whether arterial stiffening is associated with TGs in subjects is not well established. The present study investigated the associations between TGs and measures of arterial stiffness (carotid–femoral PWV and carotid–radial PWV) in a prospective community-dwelling population. We hypothesize that TGs are a predictive factor for arterial stiffness. In the present study, we examined the relationship between TGs and arterial stiffness by investigating: (1) the predictive relationship of baseline TG levels with follow-up arterial stiffness; and (2) the relationship between changes in TGs with changes in arterial stiffness in a large community-based longitudinal sample from China.

## Methods

### Subjects

This paper analysed the association between TGs and arterial stiffness (carotid-femoral PWV and carotid-radial PWV) in a community-based cohort study of subjects living in the Pingguoyuan area of Beijing, China. After a routine health check-up between September 2007 and January 2009, a total of 1680 subjects were initially eligible for cross-sectional analysis. We prospectively followed this community-based population for the first time from February 1 to September 30, 2013. Complete follow-up data were obtained from 1499 subjects (follow-up rate 89.2 %), and 181 participants were lost during the period between the initiation of the study and the follow-up. No differences other that baseline risk factors were noted in those who completed baseline and follow-up assessments. Of these, 52 were excluded from analyses because of death; thus, 1447 participants were available for analysis. The median follow-up interval for the original 1447 subjects was 4.8 years. During these visits, all participants received a questionnaire survey. Demographic information, a medical history, blood pressure measurements, and anthropometric measurements were obtained. Fasting blood and urine samples were also collected. The study was approved by the ethics committee of the People’s Liberation Army General Hospital, and each subject provided informed written consent.

### Clinical data collection

Participants completed self-reporting standardized questionnaires about lifestyle factors, prevalent diseases, family history, and medication use. Trained medical doctors evaluated anthropometrics. Height (cm) was measured using a wall-mounted measuring tape, and weight (kg) was measured using a digital scale without shoes. Systolic and diastolic blood pressures (SBP and DBP) were measured on the right arm twice in a sitting position after 5 min of rest.

### Biomarker variable determination

Blood samples were collected from participants after overnight fast. Concentrations of fasting blood glucose (FBG), total cholesterol (TC), TGs, HDL-C, LDL-C were measured by the Roche enzymatic assays (Roche Diagnostics GmbH, Mannheim, Germany) on a Roche autoanalyzer (Roche Diagnostics, Indianapolis, IN, USA). All testing was performed in the same laboratory by well trained personnel following the criteria of the World Health Organization Lipid Reference Laboratories.

### Assessment of arterial stiffness

All subjects were asked to avoid smoking, alcohol and caffeine for at least 12 h before performing the assessment. Arterial stiffness was measured by automatic carotid-femoral and carotid-radial PWV measurements using a Complior SP device (Createch Industrie, France) after the participants had rested for 5 to 10 min in a supine position, in the morning, and at a stable temperature. PWV was measured with two strain-gauge transducers along the artery. The procedure was performed using a TY-306 Fukuda pressure-sensitive transducer (Fukuda Denshi Co, Japan) that is fixed transcutaneously over the course of a pair of arteries separated by a known distance; the carotid, femoral and radial arteries (all on the right side) were used. One transducer was positioned at the base of the neck over the common carotid artery, and the other was positioned over the femoral artery. PWV was calculated from the measurement of the pulse transit time and the distance travelled by the pulse between the two recording sites (measured on the surface of the body in metres) according to the following formula: PWV (m/s) = distance (m)/transit time (s) [13]. All baseline and follow-up measurements were performed by the same specific technicians.

### Definition of variables

Smoking status was defined as smoking 1 or more cigarettes per day for at least 1 year. Non-HDL-C levels were calculated by the following equation: TG (mmol/L) - HDL-C (mmol/L). Body mass index (BMI) was calculated by the following equation: weight (kg)/height^2^ (m^2^). Waist–hip ratio was calculated by the following equation: waist circumference (cm)/hip circumference (cm). The estimated glomerular filtration rate (eGFR) was calculated using the following Chronic Kidney Disease Epidemiology Collaboration equation: eGFR = 141 × min (Scr/κ,1) ^α^ × max (Scr/κ, 1)^-1.209^ × 0.993^Age^ × 1.018 [if female] × 1.159 [if black], where Scr is plasma creatinine (mg/dL), κ is 0.7 for females and 0.9 for males, α is −0.329 for females and −0.411 for males, min indicates the minimum of Scr/κ or 1, and max indicates the maximum of Scr/κ or 1. Hypertension was defined as a mean SBP ≥140 mmHg, mean DBP ≥90 mmHg, both, or the use of antihypertensive medication. Diabetes mellitus (DM) was defined as a fasting glucose ≥7.0 mmol/L, glucose ≥11.1 mmol/L at 2 h after an oral 75 g glucose challenge, the use of antihyperglycaemic medication, or both.

### Statistical analyses

The characteristics were expressed as the median (interquartile range) or mean ± standard deviation (SD) for continuous variables and percentages for dichotomous variables. Follow-up carotid-femoral PWV was defined as elevated (≥12 m/s) or normal level (<12 m/s) [8]. Differences in the baseline levels of risk factors and clinical characteristics between subjects with elevated and normal carotid-femoral PWV over 4.8 years of follow-up were analysed using a *t*-test for continuous variables and a chi-square test for categorical variables.

The Pearson correlation was used to describe the correlations between the baseline TG level and follow-up arterial stiffness. Multiple linear regression analysis were performed to evaluate the associations between baseline TG levels and follow-up arterial stiffness (dependent variables: carotid-femoral PWV or carotid-radial PWV as a continuous variable; independent variables: age, gender, hypertension, DM, current smoking, levels of plasma TGs, non-HDL, LDL-C, SBP, DBP, FBG, BMI, weight, waist, waist–hip ratio and eGFR). As necessary, TG levels and other biomarkers were normalized by natural logarithm transformation.

We investigated the association of change in TG levels with the change in arterial stiffness (carotid-femoral PWVδI vs. carotid-femoral PWVδII; carotid-radial PWVδI vs. carotid-radial PWVδII) with logistic regression models. The change in TG levels was expressed as TGδ (TG_follow-up_-TG_baseline_). The change in arterial stiffness was expressed as PWVδ (PWV_follow-up_-PWV_baseline_). PWVδ was categorized as PWVδI (PWV_follow-up_-PWV_baseline_ <0) and PWVδII (PWV_follow-up_-PWV_baseline_ ≥0). Forward stepwise multivariate logistic regression analysis was performed to evaluate odds ratios (OR) and 95 % confidence intervals (CI). Regression models were adjusted for age and gender (model 1) as well as hypertension, DM, current smoking, change in TGs, change in non-HDL-C, change in LDL-C, change in SBP, change in DBP, change in BMI, change in weight, change in waist, change in waist–hip ratio and change in eGFR (model 2).

Receiver operating characteristic (ROC) curves were used to assess the ability of the baseline TG level indices to predict arterial stiffness assessed by carotid-femoral PWV and carotid-radial PWV.

All analyses were conducted using SPSS software for Windows, version 13.0 (SPSS, Chicago, IL, USA). We used Bonferroni correetion for multiple testing. *P*-values <0.05 were considered statistically significant.

## Results

### Clinical characteristics of the subjects categorized by gender

Altogether, we included 1447 subjects in the present study. The baseline characteristics of the study population according to carotid-femoral PWV groups (elevated or normal) are summarized in Table 1. The mean age (±SD) in the study was 61.30 ± 11.4 years. Older age, male gender, hypertension, DM, CHD, current smoking, higher SBP, higher waist, higher waist-hip ratio, higher FBG, higher TG and LDL-C levels, and lower eGFR levels were significantly associated with elevated carotid-femoral PWV.

**Table 1:** Baseline characteristics of the subjects

Variables	All subjects	Carotid-femoral PWV	Carotid-femoral PWV	*P*-value
	(*n* = 1447)	≥12 (*n* = 571)	<12 (*n* = 876)	
Age	61.30 ± 11.4	65.59 ± 9.03	54.14 ± 10.11	<0.001
Male (%)	601 (41.53)	270 (47.28)	331 (37.78)	<0.001
BMI	25.41 ± 3.32	25.41 ± 3.28	25.26 ± 4.17	0.583
SBP (mmHg)	128.7 ± 17.7	135.38 ± 18.37	124.37 ± 15.93	<0.001
DBP (mmHg)	77.11 ± 10.26	77.00 ± 10.92	77.18 ± 9.82	0.762
FBG (mmol/l)	5.39 ± 1.65	5.63 ± 1.87	5.21 ± 1.35	<0.001
TG (mmol/l)	1.80 ± 1.24	1.92 ± 1.27	1.74 ± 1.24	0.017
TC (mmol/l)	5.01 ± 0.93	5.02 ± 0.97	5.01 ± 0.89	0.845
HDL-C (mmol/l)	1.34 ± 0.42	1.30 ± 0.43	1.37 ± 0.41	0.247
LDL-C (mmol/l)	2.91 ± 0.71	2.96 ± 0.73	2.87 ± 0.69	0.030
Non-HDL-C (mmol/l)	3.53 ± 1.09	3.54 ± 1.16	3.52 ± 1.06	0.730
Waist (cm)	86.45 ± 9.34	88.63 ± 9.12	85.02 ± 10.19	<0.001
Waist-hip ratio	0.87 ± 0.05	0.88 ± 0.06	0.86 ± 0.0.07	<0.001
eGFR (ml/min)	94.2 ± 14.30	88.18 ± 14.19	98.63 ± 12.45	<0.001
Smokers	380 (26.26)	175 (30.65)	205 (23.40)	<0.001
Hypertension	755 (52.17)	418 (73.20)	337 (38.47)	<0.001
New	143 (9.88)	62 (10.86)	81 (9.24)	0.360
Anti-drug	399 (52.85)	228 (54.54)	171 (50.74)	0.333
Diabetes	302 (20.87)	183 (24.22)	119 (18.45)	<0.001
New	117 (8.09)	54 (9.46)	63 (7.19)	0.148
Anti-drug	126 (41.72)	85 (46.44)	41 (34.45)	0.038
CHD	175 (12.09)	106 (18.56)	69 (7.88)	<0.001
New	94 (6.49)	45 (7.88)	49 (5.59)	0.084

*BMI* body mass index, *SBP* systolic blood, *DBP* diastolic blood pressure, *FBG* fast blood glucose, *TG* triglyceride, *TC* total cholesterol, *HDL-C* high- density lipoprotein cholesterol, *LDL-C* low-density lipoprotein cholesterol, *eGFR* estimated glome-rular filtration rate, *CHD* coronary heart disease, *PWV* pulse-wave velocity

### Association of baseline TG with follow-up arterial stiffness

**Fig. 1:**
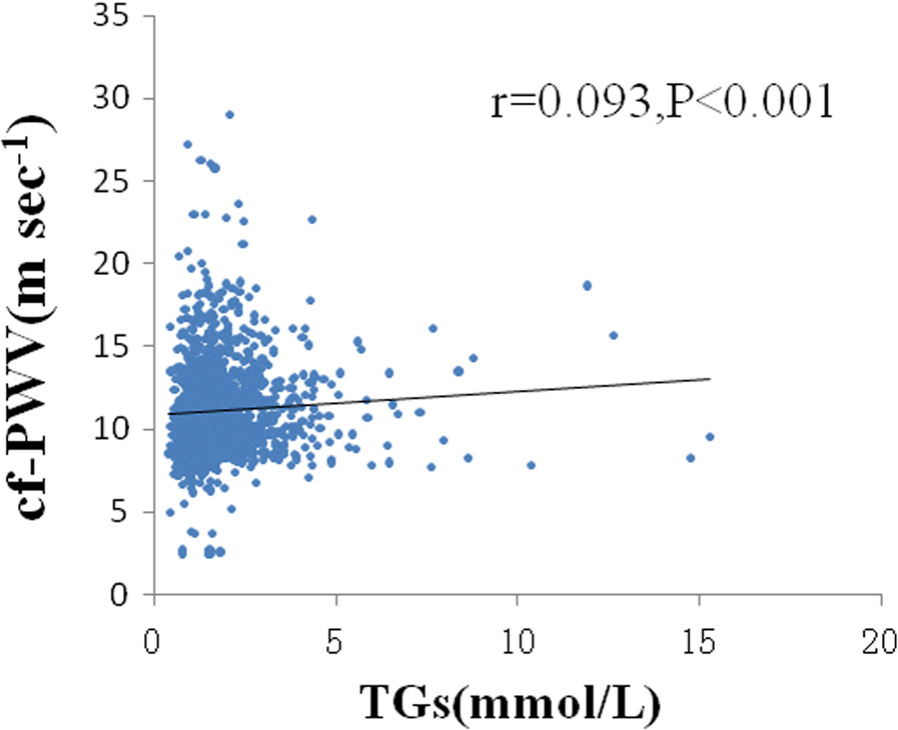
Relation between TGs and cf-PWV. The Pearson’s correlation was used to describe the relationships between TGs and cf-PWV. cf-PWV was positive relationship with TGs in 1447 subjects. cf-PWV, carotid–femoral pulse wave velocity; TGs, Triglycerides; X-axis: the value of TGs (mmol/L); Y-axis: the value of cf-PWV (ms^−1^); r, coefficient of Pearson’s correlation; *P* < 0.001 with statistical significance

Age (*r* = 0.528; *P* < 00.001), SBP (*r* = 0.325; *P* < 0.001), Waist (*r* = 0.187; *P* < 0.001), waist –hip ratio (*r* = 0.084; *P* = 0.002), FPG (*r* = 0.129; *P* < 0.001) and TGs (*r* = 0.093; *P* = 0.001, Fig. 1) were significantly and positively related to carotid-femoral PWV, but DBP (*r* = −0.003; *P* = 0.904), weight (*r* = 0.007; *P* = 0.787), BMI (*r* = 0.011; *P* = 0.687), TC (*r* = 0.020; *P* = 0.457) and (*r* = 0.033; *P* = 0.233) were not. Non-HDL-C (*r* = − 0.090; *P* = 0.001) and eGFR (*r* = −0.384; *P* < 0.001) were inversely related to carotid-femoral PWV.

**Fig. 2:**
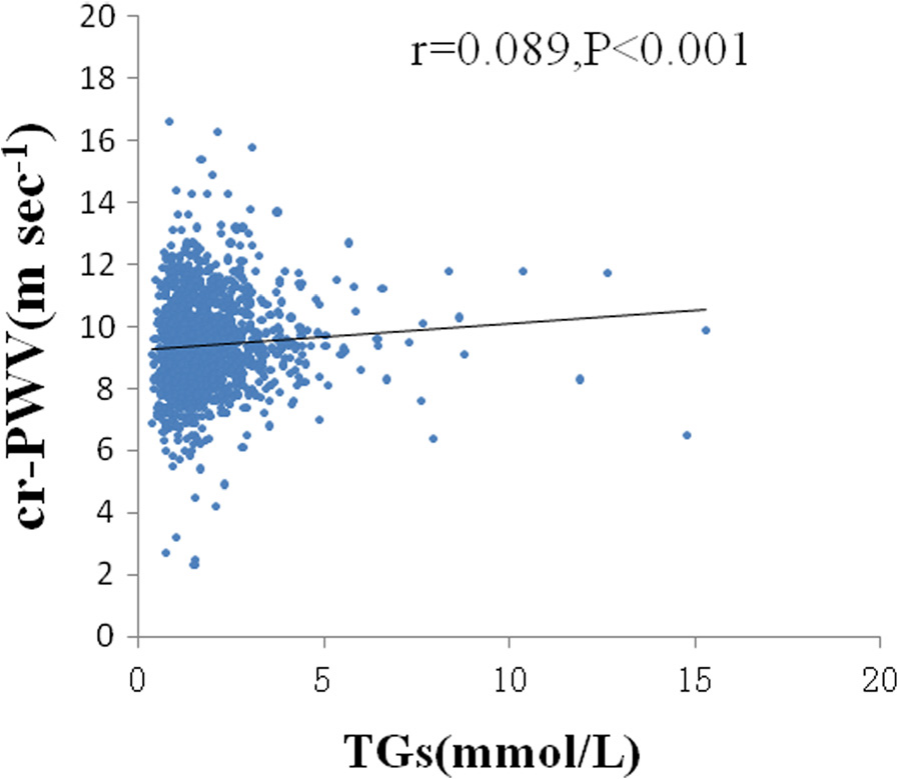
Relation between TGs and cr-PWV. The Pearson’s correlation was used to describe the relationships between TGs and cr-PWV. cr-PWV was positive relationship with TGs in 1447 subjects. cr-PWV, carotid–radial pulse wave velocity; TGs, Triglycerides; X-axis: the value of TGs (mmol/L); Y-axis: the value of cr-PWV (ms^−1^); r, coefficient of Pearson’s correlation; *P* < 0.001 with statistical significance

SBP (*r* = 0.102; *P* < 0.001), DBP (*r* = 0.209; *P* < 0.001), weight (*r* = 0.171; *P* < 0.001), waist (*r* = 0.068; *P* = 0.016) and TGs (*r* = 0.089; *P* < 0.001, Fig. 2) were significantly and positively related to carotid-radial PWV, but age (*r* = 0.168; *P* = 0.245), BMI (*r* = 0.032; *P* = 0.238), waist–hip ratio (*r* = 0.082; *P* = 0.159), TC (*r* = 0.037; *P* = 0.180) and LDL-C (*r* = 0.062; *P* = 0.0.025) were not. Non-HDL-C (*r* = − 0.073 = 0.007) was inversely related to carotid-radial PWV.

The association between baseline TGs as a continuous variable (natural logarithm transformed) and follow-up arterial stiffness are summarized in Table 2. In multivariable linear regression analysis, baseline TG levels were positively and independently associated with follow-up carotid-femoral PWV (β = 0.747, *P* < 0.001) and carotid-radial PWV (β = 0.367, *P* = 0.001), respectively. Furthermore, non-HDL-C, hypertension, diabetes (β = 0.623, *P* = 0.007), BMI and fasting blood glucose were positively and independently associated with carotid-femoral PWV, older age, LDL-C, SBP and weight; weakly associated with follow-up carotid-femoral PWV, and eGFR; and negatively associated with follow-up carotid-femoral PWV. Being male (β = 0.456, *P* = 0.001) was positively associated with follow-up carotid-radial PWV.

**Table 2:** Multiple linear regression analysis of baseline parameters and follow-up arterial stiffness

	Carotid–femoral PWV			Carotid–radial PWV		
	β	CI	*P*-value	β	CI	*P*-value
All subjects (*n* = 1447)						
Age	0.097	0.078 ~ 0.116	<0.001	−0.025	−0.037 ~ −0.012	<0.001
Male	0.253	0.180 ~ 0.686	0.252	0.456	0.179 ~ 0.734	<0.001
Smoking	0.081	−0.261 ~ 0.424	0.641	0.049	−0.170 ~ 0.269	0.659
Diabetes	0.623	0.171 ~ 1.076	0.007	0.232	0.058 ~ 0.522	0.117
Hypertension	0.444	0.083 ~ 0.805	0.016	0.018	0.014 ~ 0.149	0.881
TG^a^	0.747	0.394 ~ 1.100	<0.001	0.367	0.140 ~ 0.593	0.002
Non-HDL-C	1.672	0.629 ~ 2.715	0.002	0.173	0.062 ~ 0.409	0.149
LDL-C	0.453	0.001 ~ 0.905	0.049	0.020	−0.269 ~ 0.310	0.892
SBP	0.040	0.028 ~ 0.052	<0.001	0.007	0.001 ~ 0.014	0.074
DBP	−0.041	−0.060 ~ −0.022	<0.001	0.012	0.001 ~ 0.024	0.047
Weight	0.037	0.006 ~ 0.068	0.021	0.005	−0.016 ~ 0.025	0.657
BMI	0.160	0.073 ~ 0.246	<0.001	0.055	0.002 ~ 0.109	0.058
Waist	0.011	0.024 ~ 0.047	0.521	0.005	0.008 ~ 0.038	0.621
Waist–hip ratio	1.876	0.946 ~ 3.698	0.140	0.331	0.038 ~ 1.169	0.790
FBG	0.127	0.021 ~ 0.234	0.019	0.051	−0.017 ~ 0.119	0.143
eGFR^a^	−1.672	−2.715 ~ −0.629	0.002	0.084	−0.584 ~ 0.753	0.754
Subjects older than 65 years (*n* = 625)						
Age	0.091	0.045 ~ 0.137	<0.001	−0.032	−0.056 ~ −0.008	0.009
Male	0.308	0.059 ~ 1.372	0.408	0.029	−0.354 ~ 0.413	0.881
Hypertension	0.409	−0.148 ~ 0.966	0.138	−0.020	−0.311 ~ 0.272	0.894
Diabetes	0.664	0.019 ~ 1.346	0.057	−0.158	−0.516 ~ 0.199	0.385
Smoking	0.097	−0.456 ~ 0.650	0.732	−0.068	−0.358 ~ 0.221	0.644
TG^a^	1.094	0.449 ~ 1.738	0.001	0.524	0.186 ~ 0.861	0.002
Non-HDL-C	1.166	−2.574 ~ 1.965	0.174	−0.202	−1.840 ~ 1.436	0.809
LDL-C	0.364	0.013 ~ 1.191	0.388	0.053	−0.380 ~ 0.486	0.810
SBP	0.042	0.025 ~ 0.059	<0.001	0.010	0.001 ~ 0.019	0.026
DBP	−0.052	−0.082 ~ −0.023	0.001	0.001	−0.015 ~ 0.016	0.928
Weight	0.056	0.004 ~ 0.109	0.036	0.034	0.007 ~ 0.062	0.002
BMI	0.249	0.103 ~ 0.394	0.001	0.123	0.047 ~ 0.200	0.002
Waist	0.039	0.020 ~ 0.079	0.507	0.002	−0.027 ~ 0.035	0.779
Waist–hip ratio	1.745	0.035 ~ 3.004	0.066	1.022	−1.988 ~ 4.561	0.541
FBG	0.148	−0.045 ~ 0.341	0.132	0.074	−0.712 ~ 0.860	0.853
eGFR^a^	−1.323	−2.631 ~ 0.242	0.083	0.063	−0.712 ~ 0.869	0.847

*TG* triglyceride, *non-HDL-C* non-high-density lipoprotein cholesterol, *LDL-C* low-density lipoprotein cholesterol, *SBP* systolic blood pressure, *DBP* diastolic blood pressure, *BMI* body mass index, *FBG* fast blood glucose, *eGFR* estimated glomerular filtration rate, *PWV* pulse wave velocity

^a^: natural logarithm transformed

§: Covariates in the multiple-adjusted models included age, gender, hypertension, DM, current smoking, levels of plasma TG、non-HDL-C、LDL-C, SBP, DBP,FBG, BMI, weight, Waist, Waist–hip ratio and eGFR

Subsequently, a subgroup analysis according to subject age was conducted (Table 2). In the group older than 65 years, the association between baseline TG levels and follow-up carotid-femoral PWV (β = 1.094, *P* = 0.001) and carotid-radial PWV (β = 0.524, *P* = 0.002) in multivariable linear regression analysis were strengthened. However, in subjects younger than 65 years, none of the follow-up arterial stiffness measures were significantly related to baseline TG.

**Fig. 3:**
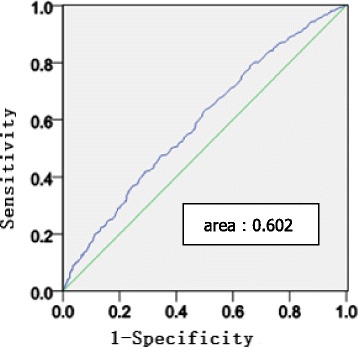
Receiver operating characteristic (ROC) curves of baseline TGs indices to predict cf- PWV. ROC analysis was performed to determine the sensitivity and specificity of the value of the area under the curve (AUC)

**Fig. 4:**
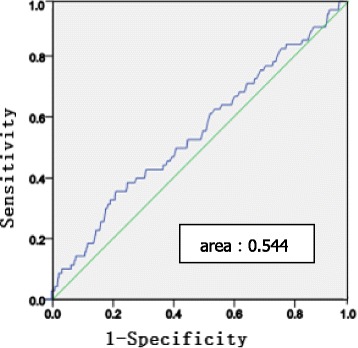
Receiver operating characteristic (ROC) curves of baseline TGs indices to predict cr- PWV. ROC analysis was performed to determine the sensitivity and specificity of the value of the area under the curve (AUC)

The receiver operating characteristic (ROC) curves for assessing the TG level indices as predictors of arterial stiffness assessed by carotid-femoral PWV and carotid-radial PWV are presented in Figs. 3 and 4.

### Effect of change in TG on change in arterial stiffness

The relationship between the change in the TG levels (TGδ) and the change in the carotid-femoral PWV (carotid-femoral PWVδI vs. carotid-femoral PWVδII) is presented in Table 3. The presence of carotid-femoral PWVδII (OR 1.107, 95 % CI: 1.039 ~ 1.299, *P* = 0.012) was significantly related to TGδ in the unadjusted model. In the adjusted models (1 and 2), the association of TGδ with carotid-femoral PWVδII remained statistically significant. Each SD increase in TGδ was associated with a 1.296-increased likelihood of the presence of carotid-femoral PWVδII (OR [per SD increase in TGδ]: 1.296; 95 % CI: 1.064 ~ 1.580; *P* = 0.010) (Model 2, Table 3). In addition, the relationship was strengthened between TGδ and the presence of carotid-femoral PWVδII (OR 1.526, 95 % CI: 1.088–2.141, *P* = 0.014) in the group older than 65 years (Model 2) but not for carotid-radial PWVδII. No association was noted in subjects younger than 65 years.

**Table 3:** Logistic regression analysis for the association between change in TG and change in carotid–femoral PWV

Carotid-femoral PWVδII	TGδ		
	OR	95 % CI	*P* Value
All subjects (*n* = 1447)			
Unadjusted	1.107	1.039 ~ 1.299	0.012
Model 1	1.180	1.041 ~ 1.396	0.033
Model 2	1.296	1.064 ~ 1.580	0.010
Subjects older than 65 years (*n* = 625)			
Unadjusted	1.312	1.057 ~ 1.745	0.002
Model 1	1.409	1.045 ~ 1.899	0.025
Model 2	1.526	1.088 ~ 2.141	0.014

*TG* triglyceride, *TGδ* change in TG, *PWV* pulse wave velocity, *PWVδII* PWV_follow-up_-PWV_baseline_ ≥ 0, *OR* odds ratio, *CI* confidence interval

model 1: age and gender

model 2: age, gender, hypertension, DM, current smoking, baseline carotid-femoral PWV, change in TG, change in non-HDL-C, change in LDL-C, change in SBP, change in DBP, change in BMI, change in weight, change in Waist, change in Waist–hip ratio and change in eGFR

## Discussion

This is the first study to observe the relationship between levels of TGs and carotid-femoral PWV in a community-based prospective sample. In the present longitudinal study, we found an association between baseline TGs and follow-up arterial stiffness independent of age, gender and other vascular risk factors, primarily in the oldest subjects. We also identified an association between the change in TGs and the change in carotid-femoral PWV, which indicates that achieving low TG levels may be an additional therapeutic consideration in subjects with atherosclerotic disease.

Several large statin trials and meta-analyses have demonstrated a reduction in LDL-C and cardiovascular morbidity and mortality. Some trials have also highlighted the significance of residual cardiovascular risk after treatment of LDL-C to target levels. Even at the LDL-C goal, patients with cardiometabolic abnormalities remain at high risk of cardiovascular events. This residual risk is partially due to high TG levels despite achievement of LDL–C goals with statin therapy. Evidence from prospective studies of the TG association supports a stronger association with CVD risk in people with lower levels of HDL-C [1, 2], higher LDL-C [1, 2] and T2DM [3, 4]. In addition, few studies have reported the association of TG levels with arterial stiffness, and the results were controversial. Henna et al. found an association between TG and arterial stiffness index in non-pregnant women even after adjustment for HDL-C and other risk factors [14]. Recently, a study [15] including 537 subjects found that TG levels were significantly associated with arterial stiffness as measured by brachial-ankle PWV in both genders. Dabelea et al. did not identify a significant relationship between baseline TG levels and arterial stiffness as measured by PWV over time, whereas they reported that an increase in TG levels of 48 mg/dL resulted in a 1.0 % increased PWV (*P* = 0.0483) [16].

The discrepancies among previous studies can be attributed to the following factors. First, the study subjects were different. The present study (the average TG level was 1.80 ± 1.24 mmol/L) was based on a community sample in which selection biases were inherently low, unlike previous studies that selected subjects with metabolic syndrome or diabetes [15, 16]. Second, our study population is relatively old, with approximately half of the subjects being ≥65 years of age. The steepest rise of transmural pressure-induced arterial wall damage occurs after the age of 60 years [17–19], leading to the strengthening of ill effects by other risk factors in older subjects. In addition, elderly subjects had an increased presence of other vascular risk factors, which may increase the susceptibility to the influence of arterial stiffness via interaction with TGs [20]. Third, the measurement of arterial stiffness was different. Arterial stiffness was measured by brachial-ankle PWV in previous studies and response of central and peripheral carotid-femoral PWV, which has no predictive value in patients with end-stage renal disease (ESRD) [21]. In the present study, arterial stiffness was assessed via the measurement of carotid-femoral PWV, which is a direct measurement of the central artery with the largest amount of epidemiological evidence for its predictive value of CV events [9]. Fourth, the present study repeatedly measured risk factors (e.g., TGs) and each participant’s outcome (e.g., arterial stiffness) that would change with time, unlike previous studies based on cross-sectional design, which could not determine whether TGs are a predictive factor for arterial stiffness.

The main finding of this study was that a higher level of TGs was an independent predictor of carotid-femoral PWV. Several potential mechanisms support TGs as a biomarker of carotid-femoral PWV risk given the role of TG-rich lipoproteins. Following the hydrolysis of exogenously derived chylomicrons or endogenously secreted very-low-density lipoproteins, cholesterol-enriched remnant by-products enter the subendothelial space [22]. Indirectly, elevated TGs impairs the capacity of high density lipoprotein to deliver cholesteryl esters, which may promote atherosclerosis via the scavenger receptor class B Type I (SR-BI) [23]. Moreover, cross-sectional studies [24–27] found that enhanced arterial stiffness in hypertriglyceridaemic states might be partly attributed to triglyceride-related LDL atherogenicity, such as small, dense LDL particles and oxidative modification of LDL. Hypertriglyceridemia-induced proinflammatory and oxidative milieu may further enhance adhesion molecule expression, increase foam cell formation, and increase the toxicity of smooth muscle [28, 29]. Hypertriglyceridemia also stimulates the release and/or expression of endothelial mediators in vitro, such as endothelin-1, which significantly promotes endothelial dysfunction, a critical early step in the development of arteriosclerosis [30–32].

Additionally, we found that change in TGs was associated with change in carotid-femoral PWV among subjects, indicating that lower plasma TG levels were associated with decreases in carotid-femoral PWV. The result was consistent with those reported by previous studies. As previously shown, decreased TG levels were independently associated with a reduced risk of CHD events compared with stable high triglyceride levels [33]. Moreover, in the PROVE IT-TIMI 22 trial, on-treatment TGs <150 mg/dl dramatically affects CHD events after adjustment of LDL-C levels [22]. These data lend support to the concept that achieving low TG levels may be an additional therapeutic consideration in subjects with atherosclerotic disease. Furthermore, we also observed that change in TGs was positively associated with change in carotid-femoral PWV but not with change in carotid-radial PWV. This effect may be attributed to the different morphology, material properties, and mechanical behaviour of the arterial wall [34–36]. In an experimental study of minipigs, atherosclerotic lesions were structurally heterogeneous, anisotropic, and incompressible [37]. All of these results can be explained by the theory that the composition of arterial wall material (smooth muscle cells and extracellular matrix, mainly collagenous tissue) is a strong determinant of PWV and extent of atherosclerosis [38].

In this study, we also found that age, hypertension, diabetes, non-HDL-C, FBG and BMI were positively and independently associated with carotid-femoral PWV. These results are consistent with those reported by previous studies [39]. The association between BMI and carotid-femoral PWV can be attributed to the following factors. First, insulin resistance, which accompanies abdominal obesity, has vascular effects through associated hyperinsulinaemia and increased glycaemia [40, 41]. Second, abdominal obesity might contribute to carotid-femoral PWV through inflammation [42].

The present study has limitations. First, a significant proportion of subjects (181, 10.7 %) were excluded due to loss to follow-up. This loss is a well-known and unavoidable limitation of epidemiological studies, which may be biased towards the null hypothesis. Second, the present study was based on subjects from Beijing communities; therefore, the conclusions may not represent Chinese individuals from other ethnic groups.

## Conclusion

Lower triglyceride levels were significantly associated with decreases in carotid–femoral PWV, indicating that achieving low TG levels may be an additional therapeutic consideration in subjects with atherosclerotic disease.

## Ethics approval and consent to participate

The study was approved by the ethics committee of the People’s Liberation Army General Hospital, and each subject provided informed written consent.

## Abbreviations

cf-pwv: arotid–femoral pulse wave velocity
cr-pwv: carotid–radial pulse wave velocity
LDL-C: low-density lipoprotein cholesterol
TC: total cholesterol
TGs: triglycerides
CHD: coronary heart disease
HDL-C: high-density lipoprotein cholesterol
FBG: fasting blood glucose
eGFR: estimated glomerular filtration rate
BMI: body mass index
DM: diabetes mellitus
SBP: systolic blood
DBP: diastolic blood pressure
ESRD: end-stage renal disease
SR-BI: scavenger receptor class B Type I
